# Knowledge and use of art therapy for mental health treatment among clinical psychologists

**DOI:** 10.1371/journal.pone.0303246

**Published:** 2024-05-09

**Authors:** Eugenia Priscilla Doku Asare, Sandra Boatemaa Kushitor, Edward Kofi Sutherland, Millicent Ofori Boateng, Stephen Manortey

**Affiliations:** 1 Department of Community Health, Ensign Global College, Kpong, Ghana; 2 Department of Food Science and Centre for Sustainability Transitions, Stellenbosch University, Stellenbosch, South Africa; 3 Center for Global Surgery, University of Utah Health, Salt Lake City, Utah, United States of America; University of Ghana, GHANA

## Abstract

**Background:**

Art therapy allows people to express feelings about any subject through creative work. It is beneficial for people who feel out of touch with their emotions. In Ghana, little is known about art therapy as a therapeutic tool. Herbal treatment, biomedical and faith healing practices are the most common treatment options for mental health. This research aimed to provide new insights into clinical psychologists on their knowledge and use of art therapy in treating clients and identified the enablers and barriers in this therapeutic intervention.

**Method:**

Twenty-one clinical psychologists were sampled using the snowball sampling method. They were interviewed over the phone using a semi-structured interview guide which was developed based on the predefined study objectives. Thematic analysis was employed to analyze the data resulting in three central thematic areas.

**Results:**

Twelve of the clinical psychologists were females and eight were male, with an age range between twenty-five to fifty years. The major themes identified were knowledge of art therapy, the use of art therapy and enablers and barriers in using art therapy. The study revealed that clinical psychologists had limited knowledge of art therapy mainly due to lack of training. With the use of art therapy, the participants revealed that they had used some form of art therapy before and they perceived art therapy to be effective on their clients however, they demonstrated low confidence in using it. Practitioner training and the availability of art therapy-related resources were identified as both facilitators and hindrances to the use of art therapy.

**Conclusion:**

Clinical Psychologists are cognizant of art therapy albeit they have limited knowledge. Therefore, training in how to use art therapy and the availability of resources to facilitate art therapy can be provided for Clinical Psychologists by the Ghana Mental Health Authority.

## Introduction

Common mental health disorders (CMHD) are increasing worldwide [1]. Due to demographic changes, there has been a 13% rise in mental health and substance use disorders in the last decade. Individuals with severe mental health disorders die prematurely, as much as two decades early [1]. CMHD can affect all areas of life, such as school or work performance, relationships with family and friends, and the ability to participate in the community. For example, two of the most common mental health disorders, depression, and anxiety cost the global economy US$ 1 trillion each year [2]. Although pharmacological treatment is the first choice for treating mental health disorders, antipsychotic medications have adverse side effects that can reduce the quality of life [3]. Furthermore, adherence to antipsychotic medication is low as reported among people with mental disorders, with schizophrenia and bipolar disorder among others [4, 5].

Therefore, Hu Y. *et al*. (2021) recommended art therapy as one of the non-pharmacological interventions for treating CMHDs [6]. According to the American Art Therapy Association (2021), art therapy integrates mental health and human services by using active artmaking, creative process, applied psychological theory, and human experience which are suitable for people of all ages [7]. Some examples of art therapy include drawing, painting, dance, music, ceramics and etc. Raffaelli (2012) included that drawing is one of the approaches that is popular with and widely used by the art therapy community [8]. One of the main goals of art therapy is to improve people’s well-being and functioning capabilities. There has been reports on the effectiveness of art therapy in some context. Robinson *et al*. (2021) also suggested that art therapy is not just essential for children but adults can also reap a lot of health benefits throughout life [9]. According to UK professionals in the field of art therapy, the key benefits of art therapy were the clients’ ability to express themselves verbally and artistically, providing evidence to suggest that therapists were confident and cognizant with art therapy [10]. Westrhenen *et al*. (2019) also determined whether the use of creative arts in a group psychotherapy program for kids might impact their post-traumatic stress disorder symptoms, behavioural issues, and post-traumatic growth (PTG). The authors reported that art therapy was helpful to re-establish or build appropriate emotion regulation after experiencing extreme stress by decreasing hyperarousal symptoms [11]. The study added that South Africa is the first and only country in the African continent to offer art therapy as a course in their degree program at the University of Johannesburg [11].

In Ghana, mental health disorders are a leading cause of years lived with disability [12]. About 13% of the adult population is estimated to be affected by mental health disorders of varying forms and may require either pharmacological or non-pharmacological care [13]. According to Kpobi and Swartz (2019), mental health treatment in Ghana involves a combination of biomedical, indigenous and faith-healing approaches. Even though 20% of patients had sought help from alternative healers for the first episode of illness, the majority of patients sought help from biomedical facilities despite the assumed supernatural illness beliefs [15]. Medical pluralism comes with its own challenges such as mutual distrust, power differentials, conceptual and methodological problems, and a lack of organizational support and resources [14], Ursula *et al*. (2023) suggest that community engagement, dialogue, and mutual learning may enable more effective and sustainable collaboration between ethnomedicine and biomedicine [14]. The Ghana Health Service provides mental health care through psychiatrists and clinical psychologists. The perceived high cost of biomedical services is one key factor that affects the treatment [15].

Ghana is a country where art thrives [16]. With a strong history of traditional modern art types, art has also been a part of Ghanaian culture. For example, through kente weaving, beads making, adinkra symbols, textiles, music, ceramics, basket weaving and theatrical plays among others. Ghana has several art galleries that exhibits paintings and sculptures to the public at the regional and community levels. Some of these art galleries include Arts Alliance Ghana Limited, Wild Geeko, Nubuke Foundation, the Aburi Craft Center, Loom and Endrose African Art Gallery. Ghana is also known for the Chalewote festival, a street art festival that celebrates the rich art culture of Ghana [17].

Despite, Ghana’s rich art culture, only three studies were noted to have examined the use of art therapy in mental health treatment in Ghana [18–20]. According to Ndaa *et al*. (2021), the process of creating and expressing one’s thoughts in beading can elevate the person’s sense of consciousness and transform the mind into a place of healing, peace, and creativity [18]. In another study, painting skills offered clients a channel to express their feelings [19]. Art therapy and its potential in healing persons dealing with psychological challenges in a Ghanaian prison environment was examined and it was recommended that the Ghana Prison Services should incorporate art therapy into their psychotherapeutic sessions in order to manage undesirable psychological issues in prisons [20]. However, a meeting with the Mental Health Authority in 2023 confirmed that there are no policies on the use of art therapy in Ghana. Since the previous studies in Ghana have recommended the use of art therapy for individuals with mental disorders, a gap remains regarding the capacity of clinical psychologists to provide this type of therapy.

As stigma has the power to prevent people from seeking help because they are seen to be weak and unable to deal with their own emotions, art therapy as a therapeutic process is achieved through art making, making it easier for people to seek help in that regard. Creating that awareness on the benefits of art therapy with this study would help to understand what clinical psychologists know about art therapy. In addition, it will inform whether they use any form of art therapy and whether it is beneficial to people with mental disorders. Furthermore, it can help to identify treatment challenges in using art therapy, the enablers and how it can be incorporated as part of treatment services since evidence in Ghana has shown improvement in people with mental disorders. Thus, this current study assessed the knowledge and use of art therapy amongst clinical psychologists as a therapeutic tool in Ghana.

## Materials and methods

### Study design and setting

This study employed a qualitative study design in Accra, Ghana. Ghana is located on the Gulf of Guinea in Western Africa. The country’s capital and largest city in terms of population is Accra which is situated on the Atlantic coast and has an urban population of almost three million [21]. Accra is the economic hub and increasingly popular tourist destination [21]. Accra is associated with higher rates of common mental health problems compared to rural areas: almost 40% risk of depression, 20% risk of anxiety and 10% risk of schizophrenia [22]. The impact of urbanization is associated with an increase in mental disorders [23]. The reason is that the movement of people to urban areas needs more facilities to be made available and infrastructure to grow. This does not happen in alignment with the increasing population, resulting in a lack of adequate infrastructure, increased risk of poverty, and exposure to environmental adversities [23]. Making some of these disorders severe when it comes to mental disorders, depression, substance abuse, alcoholism, crime, family disintegration, and alienation [23].

The country has three major psychiatric hospitals; the Accra Psychiatric Hospital, Pantang hospital and Ankaful Hospital. These facilities provide psychotherapies, Out-Patient & In-Patient Management, Teaching and Research and Occupational Therapy Services. However, the facilities are under-financed, congested, and under-staffed therefore many turns to resort to traditional or faith healing which they consider as relatively inexpensive [24]. Adherence to mental health medication is problematic in Ghana [13]. Due to the stigma surrounding mental illness, patients present late for treatment. In managing these patients, medications are mostly employed. Clinical psychologists are the main health worker cadre who provide care for mental health clients at facilities [21].

Art therapy has been used as treatment and advocacy model as well in Ghana. Through arts such as bead making, soothing and its therapeutic effects have been reported among clients. According to Ndaa (2021), the process of creating and expressing one’s thoughts in beading has the potential to elevate the person’s sense of consciousness and transform the mind to a place of healing, peace, and creativity [18]. In another study painting skills offered clients a channel to express feelings [19]. Art therapy and its potentials in healing persons dealing with psychological challenges in a Ghanaian prison environment was examined [20]. It was discovered through this research that art therapy can be utilized beyond the normal psychotherapies; Koomson recommended that the government of Ghana and the Ghana Prison Service explore and integrate art therapy as part of its psychotherapies in their efforts to manage unwanted psychological problems in prisons [20]. With these studies [18–20] showing the benefits of art therapy in improving mental illness, this current study, therefore, seeks to assess the knowledge and practices of clinical psychologists in the use and benefits of art therapy as a therapeutic tool.

### Data collection

The participants were recruited using the Ghana Psychological Council’s (GPC) 2021 Standing Register, of practising clinical psychologists [21]. With the permission of the GPA executives, the study advert was placed on their WhatsApp page to facilitate recruitment. Clinical psychologists who contacted the first author referred other clinical psychologists through a snowballing approach.

A semi-structured interview guide (ie., **S1 Appendix**) was developed for data collection based on the study objectives and literature. This allowed participants to speak about their experiences of art therapy. Art therapy was explained to them as engaging the mind, body and spirit in ways that are distinct from verbal communication by using creative media such as paper, paints, pen, collaging, music or movements like dancing in the psychological treatment of clients with CMHDs. Examples of questions that were included in the guide were *“Tell me your experience of being a clinical psychologist*?*” “Do you use any form of art therapy in your practice as a clinical psychologist*?*”* The entire data was collected via phone interviews scheduled at mutually convenient times with clinical psychologists due to their busy work schedules and unavailability for in-person interviews. Participant interviews were conducted in the English language with their prior verbal permission to record the conversation. Follow-up probe questions were used to explore the participants’ views. Interviews lasted approximately between 20-30mins. To ensure the anonymity of participants, the names of participants were not reported. Interviews were audio-recorded and transcribed verbatim for analysis.

### Data analysis

A thematic analysis approach was used employing Braun and Clark’s six phases [25]. The process of thematic analysis was done by reading the transcribed interview several times and coding relevant information. Five of the transcripts were coded to generate initial codes. Both inductive and deductive codes were used. The deductive codes included pre-determined codes such as types of art (painting, drawing, beading), and types of psychological treatment (cognitive behavior therapy, group therapy). The inductive codes were generated based on the data. Codes were shared and scrutinized until consensus was reached between PDA, SBK and MOB, after which a coding frame was generated (ie., **S2 Appendix**). The coding frame was used to code the remaining 16 transcripts. This approach is parallel with the recommendation of Creswell and Miller’s (2000), of the importance of cross-validation and group interpretation which facilitates analytic rigor and validity of the findings of qualitative studies [26, 27].

**Fig 1:**
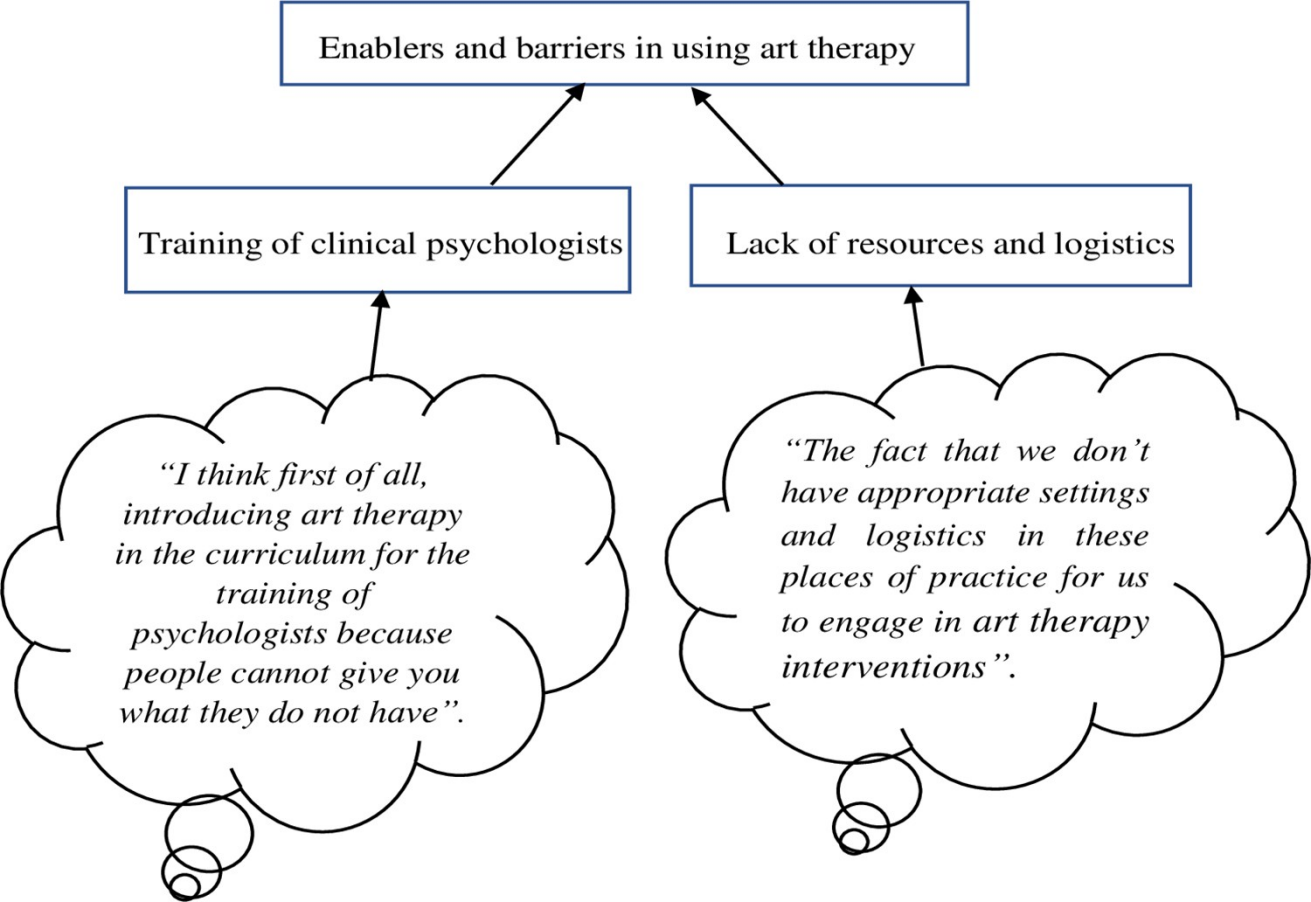
Coding tree of enablers and barriers in using art therapy for mental health treatment.

The codes were connected to generate themes. Codes that were related to a specific question were grouped together as a theme (**Fig 1**). **Fig 1** is a sample of a coding tree which shows how quotes are grouped into codes and then these codes are used to generate the themes. Finally, compelling quotes were selected from the transcripts which represented lucid elements of the working themes and were relevant to our research question [26, 28].

In this study, saturation was measured using code frequency counts and higher-order groupings [25, 29]. The coding frame designed from the first five interviews was used to code the next ten interviews. About fifteen new codes were identified. In the last set of six interviews, only three new codes were identified. To conduct the high-order groupings, the spread of codes was used as the stopping criterion. The research team agreed that if dominant codes were found among at least 15–20 of the participants, then saturation was reached. **S2 Appendix**, provide the spread of codes among the participants.

### Ethics approval and consent to participate

Ethical clearance was granted and received from the Ensign Global College’s ethical review committee (ENSIGN/IRB-GM/ET/175). Participants’ consent was sought prior to the interviews conducted with the clinical psychologists. Participation was voluntary, and the written consent information specified that the study would keep participants anonymous during data analysis and presentation.

## The participants

In all, twenty–one clinical psychologists’ were interviewed, twelve females and nine males with an age range between twenty-five to fifty years. All twenty–one had a master’s degree in clinical psychology. Their years of practice as clinical psychologists ranged between one year to twenty years and the average years of practice was six years. **Table 1** shows the demographic characteristics of the participants.

**Table 1:** Demographic characteristics of participants.

Code	Sex	Age Range	Facility type	Years of practice	University attended	Program offered
**R1**	Male	36- 40years	Government hospital(tertiary)	6 years	University of Ghana	MPhil in Clinical Psychology
**R2**	Female	31-35years	Government hospital(tertiary)	6 years	University of Ghana	MPhil in Clinical Psychology
**R3**	Female	36-40years	Government hospital(tertiary)	3 years	University of Cape Coast	MPhil in Clinical Health Psychology
**R4**	Female	31-35years	Government hospital(tertiary)	6 years	University of Ghana	MPhil in Clinical Psychology
**R5**	Female	50-55years	Government hospital(tertiary)	20 years	University of Ghana	MPhil in Clinical Psychology
**R6**	Male	45-50years	Government hospital(tertiary)	7 years	University of Ghana	MPhil in Clinical Psychology
**R7**	Female	41-45years	Private hospital & private school(tertiary)	5 years	Methodist university	MPhil in Counselling Psychology with Clinical Option
**R8**	Female	31-35years	Government hospital(primary)&private practice	10 years	University of Ghana	MPhil in Clinical Psychology
**R9**	Male	31-35years	Government hospital(primary)&research centre	5 years	University of Ghana	MPhil in Clinical Psychology
**R10**	Female	25-30years	Private preparatory school	3 years	University of Cape Coast	MPhil in Clinical Health Psychology
**R11**	Female	31-35years	Government hospital(tertiary)	11 years	University of Ghana & Stellenbosch university	MPhil in Clinical Psychology& Public Mental health
**R12**	Male	25-30years	Government hospital(primary)&private preparatory school	3 years	University of Cape Coast	MPhil in Clinical Health Psychology
**R13**	Female	31-35years	Government hospital(tertiary)	3 years	University of Ghana	MPhil in Clinical Psychology
**R14**	Female	25-30years	Private preparatory school	3 years	University of Cape Coast	MPhil in Clinical Health Psychology
**R15**	Male	31-35years	Government hospital(tertiary)	4 years	University of Ghana	MPhil in Clinical Psychology
**R16**	Male	36-40years	Government hospital(tertiary)	12 years	University of Ghana	MPhil in Clinical Psychology
**R17**	Female	25-30years	Private hospital(primary)	2 years	University of Ghana	MPhil in Clinical Psychology
**R18**	Male	36-40years	Government hospital(primary)& private hospital	7 years	University of Cape Coast	MPhil in Clinical Health Psychology
**R19**	Female	25-30years	Government hospital(tertiary)	1 year	University of Cape Coast	MPhil in Clinical Health Psychology
**R20**	Male	31-35years	Government hospital(tertiary)	4 years	University of Cape Coast	MPhil in Clinical Health Psychology
**R21**	Male	46-50years	Government school(tertiary)	20years	University of Ghana	MPhil in Clinical Psychology

## Results

The results were grouped under three major themes identified from the analysis of the interviews. **Table 2** shows the summary of the Themes and Subthemes: *Knowledge of art therapy* with four subthemes: *Definition and uses of art therapy*, *Training in the use of art therapy*, *Art therapy in Ghanaian context and Continuous Professional Development courses*. The second major theme was *the use of art therapy* with two sub-themes: *Forms of art therapy used by clinical psychologists* and *perceived effectiveness*. The third theme were the *enablers and barriers in using art therapy*.

**Table 2:** Major themes and sub-themes.

Main themes	Subthemes
1. Knowledge of Art therapy	Definition and uses of Art therapy. Training in the use of Art therapy. Art therapy in Ghanaian context Continuous Professional Development courses.
2. The use of Art therapy	Forms of Art therapy used by clinical psychologists. Perceived effectiveness.
3. Enablers and barriers in using Art therapy	Enablers of Art therapy Barriers of Art therapy

### Knowledge of art therapy

#### a. Definition and uses of art therapy

This sub-theme sought to understand the perspectives of clinical psychologists on what art therapy is and its role in their practice. Findings showed that all twenty-one participants had some knowledge of art therapy but from different perspectives. Some participants attributed art therapy to creative art, others indicated it was a form of exploration and self-expression. Art therapy was described as clients practically doing something creative to indicate how they feel. The following extract shows the understanding of one participant on art therapy likened creative arts:

“*Art therapy is using a form of art creatively to get the person to express himself or herself”*. *(R6*, *Male*, *7 years of experience)*

Two themes were identified about the uses of art therapy. Firstly, art therapy was reported as a useful tool that facilitates therapy especially when clients cannot verbalize their problems. R14 explained with an example that if a client is asked to draw, it gives the therapist an opportunity to ask questions about what the client has drawn and further understand what the client is experiencing with the drawing.

“*Using art to explore and find out the mind of an individual or themselves in instances on what they have drawn*, *it will bring up questions that will be able to help you understand what the person is going through”*. ***(R14*, *Female*, *3 years of experience)***

Secondly, some participants also held the view that art therapy is self-expressive. Self-expression is useful in therapy as clients can freely express how they feel inward through art therapy, especially when clients do not have the language or the ability to articulate what is going on with them.

“*It’s an art used to show how people express some of their emotions that they are feeling and also helps in understanding how the client has been feeling in a way that they can’t express verbally”*. ***(R2*, *female*, *6 years of experience)*.**

#### b. Training in the use of art therapy

This sub-theme explored whether clinical psychologists were taught or used art therapy during their training. Fourteen participants had no formal training in using art therapy. Art therapy was not included in their curriculum or syllabus. Seven participants had inadequate training. For these participants, art therapy was briefly mentioned during their training compared to the other therapies, which were taught extensively. Knowledge of art therapy was primary through reading and lessons from other subjects (especially performing arts).

“*No*, *it [art therapy] wasn’t part of the curriculum when I was studying psychology”*. ***(R18*, *Male*,*7 years of experience)*.**

“*I am able to apply my expertise in performing art so art therapy wasn’t part of the curriculum when I was studying psychology”*. ***(R6*, *Male*,*7 years of experience)*.**

“*Art therapy was mentioned in passing but we didn’t really delve so much into art therapy so the bases of my work*, *I have to read through art therapy”*. ***(R8*, *Female*, *10 years of experience)*.**

“*Art therapy was not one of the core therapies we were introduced to but then it was mentioned as part of the training*, *to say that*, *it wasn’t the focus of the training*, *so I actually read about art therapy just to have an idea of it*. *so yes*, *it’s my personal reading”*. ***(R20*, *Male*, *4 years of experience)***

#### c. Continuous professional development courses

The CPD course is a learning platform to maintain proficiency and competence in a profession where participants (ie., Clinical Psychologists) are awarded credit points which is accumulated to maintain good standing in their respective fields of work. CPDs provide a platform for individuals to learn new techniques, approaches and skills to manage and ensure proficiency and competency in their profession. It is mandatory to have evidence of required credit points to renew one’s license every year with the GPA. The continuing professional development course (CPD) points should be obtained from at least three (3) different levels of activities.

In addition to assessing participants’ training in art therapy, findings revealed that participants had never come across a course on art therapy in any of their Continuous Professional Development (CPD) courses.

“*In my line of practices*, *I have never come across any course or any CPD program which is related to art therapy”*. ***(R15*, *Male*, *4years of experience)***

“*No*, *I haven’t come across any course on art therapy”*. ***(R16*, *Male*, *12 years of experience)***

“*No*. *have I attended two CPD courses*, *none of them was on art therapy”*. ***(R20*, *Male*, *4years of experience)***

#### d. Art therapy in Ghanaian context

About thirteen (13) participants were of the view that art therapy in the Ghanaian context would be a cultural challenge due to low acceptance and cultural sensitivity. Some stated that clients tend to ask for medication rather than art therapy when suggested because they presume that the medication is faster than art therapy.

“Per our culture, I foresee a little challenge, so when I say a little challenge even with our normal in quote “therapies” that we are using and doing with the other theories, most clients or most people come and they would want medications, they come with their challenges and they are expecting you to give them medications”. **(R7, female, 5 years of experience).**

“*Our culture is a bit different when it comes to health-related issues like mental health disorders*, *so there are things that we don’t appreciate in the Ghanaian context”*. ***(R10*, *female*, *3 years of experience)*.**

However, this view was not supported by two participants. They were of the view that art therapy in the Ghanaian context is acceptable and should be embraced. On the issue of cultural sensitivity, a participant explained that art forms can be incorporated to suit any culture; therefore, in the Ghanaian context, art forms that are interesting to an average Ghanaian can be fitted into our culture.

“*I think Ghanaians would be open to it*, *we are a very art-conscious country so it’s not something that Ghanaians would kick against”*. ***(R11*, *female*, *11 years of experience)*.**

“*Well creativity can be bent to fit any culture and so if I am pushed to see a benefit then I can say that we can look in our context to see the sort of art forms*, *that your average Ghanaian is interested in or is able to express well in and therefore adapt it to suite our need****”*. *(R5*, *female*, *20 years of experience)*.**

### The use of art therapy

#### a. Forms of art therapy used by clinical psychologists and their perceived effectiveness

In this subtheme, participants were asked if they have used art therapy in any form. The majority, reported having used a form of art therapy such as painting, writing and music, but drawing was used mostly. Six participants indicated that they had never used it. Participants explained that they used drawings and paintings in their practice to know how a person was feeling.

“*I used painting and different drawings to understand how the person feels ok*, *I used different colors that the person can link it to his feelings and I used images to understand the persons feelings*, *I think that’s all”* ***(R12*, *male*,*3 years of experience)*.**

“*No*, *so as far as I remember I haven’t recommended it to my clients before*, *it hasn’t occurred to me to use it”*. ***(R10*, *female*, *3 years of experience)*.**

In this subtheme, participants indicated how they perceive art therapy and its effectiveness on their clients. Some used art therapy because they work with children and tend to help children open up in therapy when they started using art therapy.

“*I read about activities I could do to engage and help the children warm up to me*, *art therapy was recommended so I started it and it has worked”* ***(R14*, *Female*,*3 years of experience)***.

A participant explained that they used toys to create shapes and forms to occupy children who are hospitalized and having terminal illnesses. Art therapy was used as a form of distraction especially for kids to help cope with pain and an outlet to express themselves.

“*I use toys to help children going through terminal illnesses and painful medical experiences to help minimize the intensity of the pain and distract them from the reality of the pain*, *as well as psychological pain associated with the physical pain”* ***(R1*, *male*, *6 years of experience)*.**

### Enablers in using art therapy

This theme is about how art therapy can be facilitated and what will make clinical psychologists more likely to use art therapy in their practice. For all the participants, facilitating art therapy begins with the training of practitioners to provide them with the competence in using it. They also mentioned research, education, and provision of resources at facilities to engage the clients in art activities such as painting, art media such as canvas, art therapy books, etc. Participants suggested that art therapy can be introduced into the curriculum for Master’s Degree programs and continuous professional development (CPD) as described below:

“*I think first of all*, *introducing art therapy in the curriculum for the training of psychologists because people cannot give you what they do not have”*. ***(R9*, *male*, *5 years of experience)***

“*It could also be in terms of the CPD programs practicing psychologists can look at that in adopting it as part of their professional processes”* ***(R15*, *male*, *4 years of experience)***

“*I think first of all*, *to some extent what we are doing now is the foundation that needs to be laid*, *researching*, *finding out exactly what is out there*, *what people know and then building up on with that knowledge on training”*.***(R4*, *female*, *6 years of experience)*.**

Participants also added that clients should be educated on art so they are enlightened on the benefits of the treatment given to them so they can appreciate the progress when it starts.

“*Education is key because giving any form of therapy to any client*, *you need to educate them for them to understand the rationale behind that”*. ***(R3*, *female*, *3 years of experience)*.**

### Barriers to using art therapy

Four main barriers were reported by the participants, they are lack of art therapy skills among the clinical psychologists, logistics and perceived low acceptability of art therapy in Ghanaian culture. The majority (nineteen) of the participants were of the view that because they lacked knowledge and training, it was difficult for them to practice. Others stated the cost involved for both the client and the therapies, a lack of skills and resources and time-consuming nature and the lack of appreciation of art therapy. Some participants were of the view that the lack of training prevents them from managing clients with art-related activities. Even though the client might have an interest in it, they would not know exactly what to do.

Well training definitely, if the people are not trained to use it, they wouldn’t know what to do and it’s appropriate that they know the right thing to do”. **(R5, female, 20 years of experience)**

In terms of logistics, the participants were concerned about the potential cost of the therapy and the lack of space to practice art therapy

“*The fact that we don’t have appropriate settings and logistics in these places of practice for us to engage in art therapy interventions”*. ***(R5*, *female*, *20 years of experience)*.**

“*We can never talk about barriers without bringing economics*, *many patients are unable to afford art therapy because its expensive”*. ***(R1*, *male*, *6 years of experience)*.**

## Discussion

This study assessed the knowledge and the use of art therapy amongst clinical psychologists in treating people with mental disorders. This section discusses the major themes identified in the study by comparing the findings to other studies in different contexts. The majority of the participants were females although an almost equal number to males. It’s a wide held stereotype that psychologists are mostly female, however the trend is changing. Females are more drawn to psychology because they perceive themselves as more empathic than men [30]. A study done by Crothers *et al*. (2010) showed the factors related to gender-based differences include a greater likelihood that women tend to take time off to have or care for children, take family leave or work part-time, and work in non-profit or local government sectors compared to males. With these findings, many women are drawn to the flexibility that a career in psychology provides [31].

Participants included in the study had a wide range of experience in the profession and could speak about the topic on their experience with art therapy. All participants had a Master’s degree in clinical psychology. In Ghana, to qualify as a clinical psychologist, the requirement is to complete an MPhil in Clinical Psychology which is provided by only two public universities [21]. The majority of the participants held a degree from these two public universities.

The findings showed that the majority of the participants had inadequate knowledge and usage of art therapy. This can be explained by the limited focus on art therapy in their training as reported by the participants. As reported by Dzokoto and colleagues, psychotherapy is among the dominant treatment models in Ghana [32]. Yet, psychological treatment methods in mental health is extremely limited in most parts of the country [33]. It is possible that the lack of training and use of art therapy in mental health treatment in Ghana may be due to the cost involved in teaching and practicing art therapy. Clinical psychologists have reported lack of resources to support their wholistic training and practice. Secondly, some of the participants stated that there would be a cultural challenge due to low acceptability. Patients prefer medications rather than therapies due to the stigma surrounding mental illness, patients present late for treatment [13]. In managing these patients, medications are mostly employed [13].

Among those who used art therapy, drawing, was the dominant art form was used and mainly among children. This finding is supported by Raffaelli (2012) showed the use of collage and drawing materials are the two approaches that are popular with and widely used by the art therapy community [8]. With more exposure and training for clinical psychologists, the use of art therapy can be expanded to include adults.

Another finding was that majority of the participants revealed they have used a form of art therapy even though they were uncertain about its functionality. They reported low confidence while using art therapy because they are not trained or have no competence in art therapy. It is therefore possible that acquiring the right training and knowledge can improve confidence in using art therapy. According to Brooks (2015), confidence is one trait that has been used to measure effective learning connected with information literacy abilities [34]. Therefore, Brook’s study supports that without information or knowledge, effective learning has not taken place.

This study included only clinical psychologists in Accra. However, there may be other clinical psychologists in other parts of the country who were not involved in the study hence their views were not captured. Nevertheless, this is the first study to examine the capacity of clinical psychologists to use art therapy. This current study recommends that the Ghana Psychology Council (GPC), together with the Ghana Psychological Association (GPA), introduce additional training programs on CPD art therapy courses, offering targeted workshops or seminars with the ultimate goal of building a “culture” that values and promotes art as an essential component of training in psychology. Art therapy can be incorporated to suite our culture like traditional art forms such as kente weaving, pottery, woodcarving, sculpture, basketry, bead making etc which has been passed down through generations, preserving Ghana’s cultural identity. This research also recommends that art therapy can focus on children in our Ghanaian society since children enjoy activities and are more prone to open up more during therapy sessions.

The provision of resources was also one of the factors the findings revealed in both the barriers and enablers. This could mean that even when a clinical psychologist is trained in art therapy and the resources including logistics are not available to facilitate the use of art therapy, it would be difficult for the clinicians to recommend and apply it in their sessions [35]. These may differ depending on the form of art therapy the clinical psychologist may be applying, be it painting, drawing, pottery, writing, dancing, music, photography, reading, etc.

Each form of art therapy has its supplies and until these supplies are provided, effective therapy cannot be achieved. Some of these supplies are art sets such as colouring pencils or crayons, books, clay dough, papers, pencils, canvases, art therapy furniture, etc. which can be provided by the hospitals where they work and can serve as a motivation or warm up the idea of using it in their therapy sessions. Other resources such as the space or suitable environment for these forms of art therapy to take place are needed. A national gallery of art for the public to be inspired and scan the history of art and showcase some of the triumphs of human creativity, as well as offer full spectrum of special exhibitions and public programs freely are not available, to even discuss expertise needed in the area of art therapy. Lee (2022) agrees that the provision of resources such as art supplies will inspire works of art and even whole ways of thinking about art. He also added that materials open new forms of technique and expression that allow clients to communicate their thoughts and ideas that make art valuable and enduring [36].

## Conclusion

In conclusion, there is interest and appetite from clinical psychologists to use art therapy as one of the treatment options. The findings of this research showed that lack of training is a significant component of clinical psychologists having no competencies in art therapy. Clinical psychologists’ ability to be confident when applying art therapy was challenged therefore, they do not use it, and treating children without creative activities may cause a slow recovery in the therapeutic interventions. This research has provided evidence to support the proposition that clinical psychology curriculums in graduate schools could be revised for future planning and policy concerned with art therapy.

## Supporting information

S1 Appendix Interview guide for clinical psychologists. (DOCX)

S2 Appendix Coding frame. (DOCX)

## Abbreviations

GPA: Ghana Psychological Association
CMHD: Common Mental Health Disorder
GPC: Ghana Psychology Council
CPD: Continuing professional development
CBT: cognitive behavioral therapy
REBT: rational emotive behavior therapies
DBT: Dialectical behavior therapies
EMDR: Eye movement desensitization and reprocessingo
