# Supplementary material for: Knowledge and use of art therapy for mental health treatment among clinical psychologists
Source: PLoS One. 2024 May 9;19(5):e0303246. doi: 10.1371/journal.pone.0303246 (PMC11081332; doi:10.1371/journal.pone.0303246)
Supplement: S1 Appendix — (DOCX) [file pone.0303246.s001.docx]

**S1 Appendix**

**Interview guide for Clinical Psychologists**

**Assessment of The Knowledge and Practices of Clinical Psychologists in The Use of Art Therapy for Mental Health Treatment in Ghana.**

**Demographics**

1. Gender 🞎 Male 🞎 Female

2. Are you working in 🞎Private practise 🞎Public service 🞎NGO 🞎other ____________________

3. How many years have you been practicing as a clinical or health psychologist ______________________

4. Age 🞎 25-30 🞎31-35 🞎36-40 🞎41-45 🞎46-50

5. University attended for clinical or health psychology masters

­­­­­­­­­­­­­­­­­­­­­­­­­­__________________________________

**Knowledge of art therapy**

6. Tell me your experience of being a clinical psychologist? What are some of the therapies you use for your clients?

7. What do you know about art therapy?

8. Was art therapy part of your curriculum during your training? (If yes, ask specific courses or topics treated. If no, prompt: attended any seminar on art therapy or online class on art therapy etc)

9. About CPD (continuous professional development) do you intentionally choose courses on art therapy.

**Use and importance of art therapy**

10. Do you use any form of art therapy in your practice as a clinical psychologist? (Prompt: for examples of art therapy used)

11. What is your view on art therapy in psychology?

12. What is the place of art therapy in the Ghanaian context?

13. Is art therapy important or beneficial in therapy for patients?

14. What kind of mental disorders do you manage with art therapy?

**Enablers and barriers of art therapy**

15. What are some enablers in using art therapy in Ghana?

16. What are some barriers in using art therapy in Ghana?

17. Do you think art therapy should be included in the curriculum?

18. If an art therapy training course was introduced, would you attend?

**Closure & debriefing**

19.Do you have anything else to tell me about art therapy?

20. How did you feel about being interviewed on this topic?

21. Is there anything you wish had been done differently?

22. Do you have any questions for me?
