# Supplementary material for: Knowledge and use of art therapy for mental health treatment among clinical psychologists
Source: PLoS One. 2024 May 9;19(5):e0303246. doi: 10.1371/journal.pone.0303246 (PMC11081332; doi:10.1371/journal.pone.0303246)
Supplement: S2 Appendix — (DOCX) [file pone.0303246.s002.docx]

**S2 Appendix**

**Spread of codes**

**KNOWLEDGE OF ART THERAPY**

|  | R1 | R2 | R3 | R4 | R5 | R6 | R7 | R8 | R9 | R10 | R11 | R12 | R13 | R14 | R15 | R16 | R17 | R18 | R19 | R20 | R21 | SUM |
| --- | --- | --- | --- | --- | --- | --- | --- | --- | --- | --- | --- | --- | --- | --- | --- | --- | --- | --- | --- | --- | --- | --- |
| **Definition and uses of art therapy(subtheme)** |  |  |  |  |  |  |  |  |  |  |  |  |  |  |  |  |  |  |  |  |  |  |
| Creative art | X |  |  |  | x |  | x | x | x |  |  | x | x |  | x | x |  |  |  |  |  | 9 |
| Exploration |  |  |  |  |  |  |  |  |  |  |  |  |  | x |  |  |  |  |  |  |  | 1 |
| Self-expression |  | x |  | x |  | x |  |  |  | x | x |  |  |  |  |  |  | x |  |  | x | 7 |
| Management |  |  | x |  |  |  |  |  |  |  |  |  |  |  |  |  |  |  |  |  |  | 1 |
| Form of treatment |  |  |  |  |  |  |  |  |  |  |  |  |  |  |  |  | x |  | x | x |  | 3 |

|  | R1 | R2 | R3 | R4 | R5 | R6 | R7 | R8 | R9 | R10 | R11 | R12 | R13 | R14 | R15 | R16 | R17 | R18 | R19 | R20 | R21 | SUM |
| --- | --- | --- | --- | --- | --- | --- | --- | --- | --- | --- | --- | --- | --- | --- | --- | --- | --- | --- | --- | --- | --- | --- |
| **Training in the use of art therapy(subtheme)** |  |  |  |  |  |  |  |  |  |  |  |  |  |  |  |  |  |  |  |  |  |  |
| No formal training | x |  | x | x | x | x |  |  | x | x | x |  | x |  | x | x | x | x | x |  |  | 14 |
| Inadequate training |  | x |  |  |  |  | x | x |  |  |  | x |  |  | x |  |  |  |  | x | x | 7 |
|  | R1 | R2 | R3 | R4 | R5 | R6 | R7 | R8 | R9 | R10 | R11 | R12 | R13 | R14 | R15 | R16 | R17 | R18 | R19 | R20 | R21 |  |
| **CPD courses(subtheme)** |  |  |  |  |  |  |  |  |  |  |  |  |  |  |  |  |  |  |  |  |  |  |
| No art therapy course | x | x | x | x | x | x | x | x | x | x | x | x | x | x | x | x | x | x | x | x | x | 21 |

**THE USE OF ART THERAPY**

|  | R1 | R2 | R3 | R4 | R5 | R6 | R7 | R8 | R9 | R10 | R11 | R12 | R13 | R14 | R15 | R16 | R17 | R18 | R19 | R20 | R21 | SUM |
| --- | --- | --- | --- | --- | --- | --- | --- | --- | --- | --- | --- | --- | --- | --- | --- | --- | --- | --- | --- | --- | --- | --- |
| **Forms of art therapy used by clinical psychologists(subtheme)** |  |  |  |  |  |  |  |  |  |  |  |  |  |  |  |  |  |  |  |  |  |  |
| Used a form of art therapy | x |  | x |  | x | x |  | x | x |  | x | x |  | x |  | x | x | x | x | x | x | 15 |
| Never used a form of art therapy |  |  |  | x |  |  | x |  | x | x |  |  | x |  | x |  |  |  |  |  |  | 6 |

|  | R1 | R2 | R3 | R4 | R5 | R6 | R7 | R8 | R9 | R10 | R11 | R12 | R13 | R14 | R15 | R16 | R17 | R18 | R19 | R20 | R21 | SUM |
| --- | --- | --- | --- | --- | --- | --- | --- | --- | --- | --- | --- | --- | --- | --- | --- | --- | --- | --- | --- | --- | --- | --- |
| **Perceived Effectiveness(subtheme)** |  |  |  |  |  |  |  |  |  |  |  |  |  |  |  |  |  |  |  |  |  |  |
| Effectiveness of art therapy | x |  | x |  | x | x |  | x | x |  | x | x |  | x |  | x | x | x | x | x | x | 15 |

**ENABLERS IN USING ART THERAPY**

|  | R1 | R2 | R3 | R4 | R5 | R6 | R7 | R8 | R9 | R10 | R11 | R12 | R13 | R14 | R15 | R16 | R17 | R18 | R19 | R20 | R21 | SUM |
| --- | --- | --- | --- | --- | --- | --- | --- | --- | --- | --- | --- | --- | --- | --- | --- | --- | --- | --- | --- | --- | --- | --- |
| **Facilitators of art therapy(subtheme)** |  |  |  |  |  |  |  |  |  |  |  |  |  |  |  |  |  |  |  |  |  |  |
| training of practitioners | x | x | x | x | x | x | x | x | x | x | x | x | x | x | x | x | x | x | x | x | x | 21 |
| research | x |  |  | x |  | x |  | x | x |  | x |  | x |  | x |  |  | x | x | x | x | 12 |
| education |  | x | x |  |  | x |  |  | x |  | x | x | x | x | x |  |  | x | x | x | x | 13 |
| provision of resources | x | x | x |  | x | x | x |  |  | x |  | x |  | x |  | x | x |  |  |  |  | 11 |

**BARRIERS IN USING ART THERAPY**

|  | R1 | R2 | R3 | R4 | R5 | R6 | R7 | R8 | R9 | R10 | R11 | R12 | R13 | R14 | R15 | R16 | R17 | R18 | R19 | R20 | R21 | SUM |
| --- | --- | --- | --- | --- | --- | --- | --- | --- | --- | --- | --- | --- | --- | --- | --- | --- | --- | --- | --- | --- | --- | --- |
| **Hindrances in the use of art therapy(subtheme)** |  |  |  |  |  |  |  |  |  |  |  |  |  |  |  |  |  |  |  |  |  |  |
| lack knowledge and training, | x | x |  | x | x |  | x | x | x | x | x | x | x | x | x | x | x | x | x | x | x | 19 |
| Expensive | x |  |  |  |  |  | x | x |  |  | x | x |  |  |  |  |  |  |  |  |  | 5 |
| lack of skills and resources |  | x |  | x | x |  | x | x |  |  | x | x | x |  |  |  |  |  | x |  | x | 10 |
| Time consuming |  |  |  |  |  |  |  | x |  |  |  |  |  |  |  |  |  | x |  |  |  | 2 |
| Lack of appreciation | x |  |  |  |  |  |  |  |  |  |  |  | x |  |  |  |  | x |  |  |  | 3 |
